# Supplementary material for: Genomic Characterization and Initial Insight into Mastitis-Associated SNP Profiles of Local Latvian Bos taurus Breeds
Source: Animals (Basel). 2023 Aug 31;13(17):2776. doi: 10.3390/ani13172776 (PMC10487150; doi:10.3390/ani13172776)
Supplement: Supplementary file 1 [file animals-13-02776-s001.zip › Figure S1.pdf]

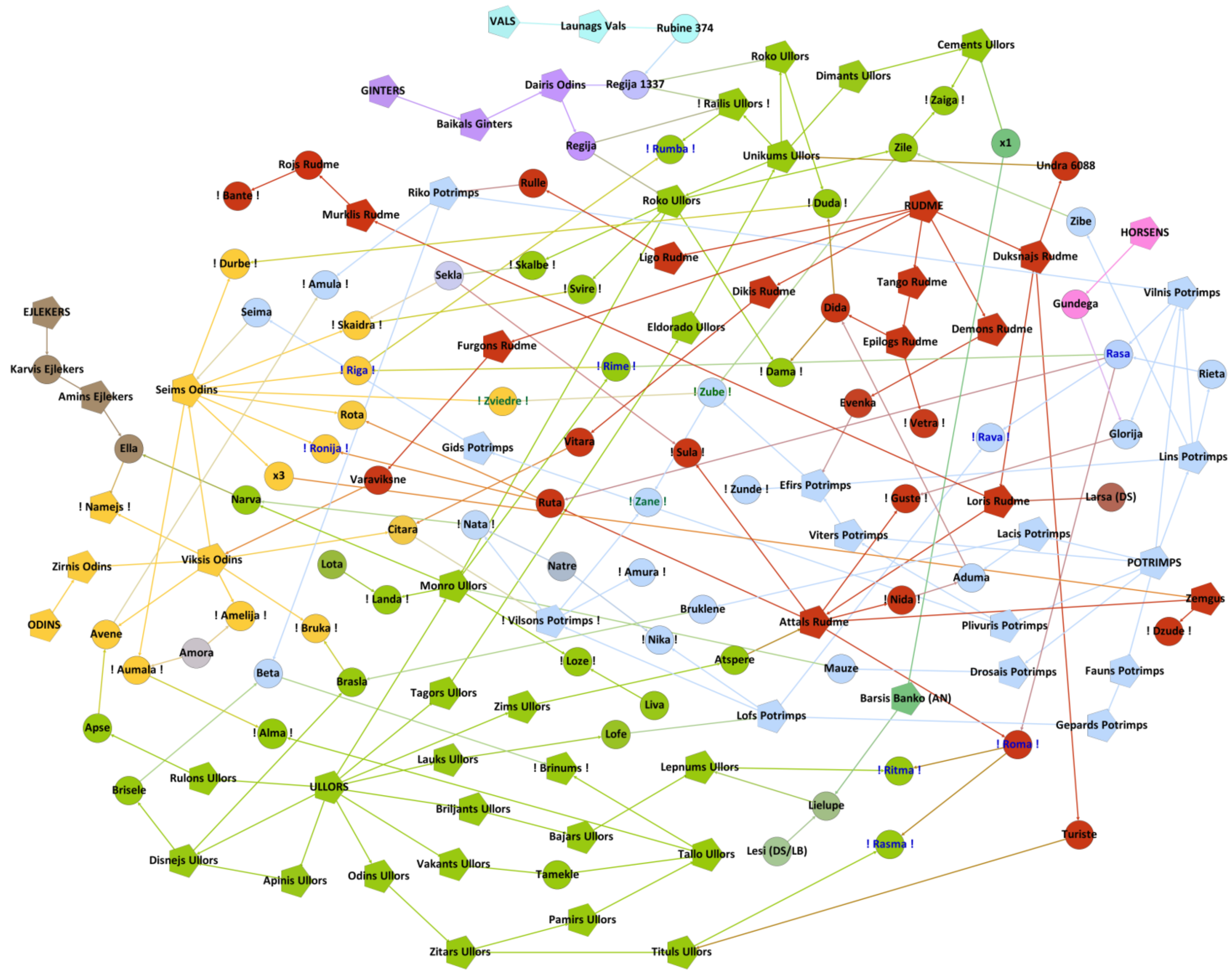

Supplementary Figure S1. Network representing kinship among LBG cattle. Dominant paternal lineages are highlighted in distinct colours and include "Ullors" with nodes coloured in green, "Potrimps" with nodes in blue, "Rudme" with nodes in red, "Odins" with nodes in yellow, "Horsens" with nodes in pink, "Ejlekers" with nodes in brown, "Ginters" and "Vals" with nodes coloured in violet and bright blue respectively. The male founder of each of the dominant lineages is denoted with capital letters. Maternal lineages with more than three cows included in sequencing analysis are indicated with names in distinct colours. Cows with shared maternal ancestry include daughters of Rasa, Rava, Roma and Rime, Rimes daughter Riga and Rasas granddaughter Ronija highlighted in dark blue, as well as Zube with her daughters highlighted in green. All sequenced animals are indicated with exclamation marks. Pentagons represent bulls and circles represent cows. "X" indicates animals with missing pedigree information. Edges show relationships between animals and are directed from ancestors towards offspring. The length of edges is determined manually to provide better resolution and has no other meaning.
