# Supplementary material for: Genomic Characterization and Initial Insight into Mastitis-Associated SNP Profiles of Local Latvian Bos taurus Breeds
Source: Animals (Basel). 2023 Aug 31;13(17):2776. doi: 10.3390/ani13172776 (PMC10487150; doi:10.3390/ani13172776)
Supplement: Supplementary file 1 [file animals-13-02776-s001.zip › Figure S10.pdf]

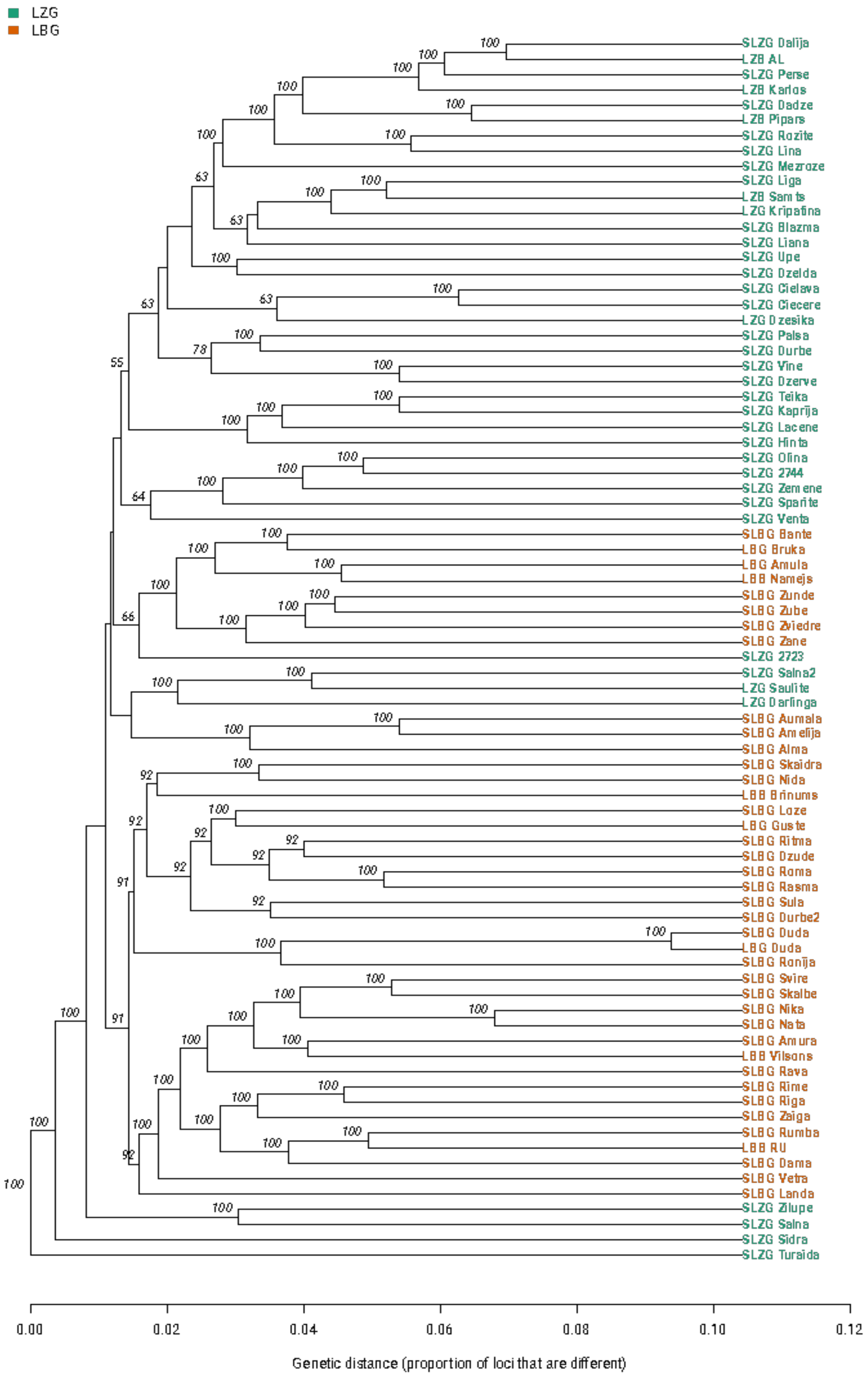

Supplementary Figure S10. Genetic distance tree based on the UPGMA algorithm representing the genetic relatedness of the LZG and LBG samples. The tree was reconstructed using 100 bootstrap replicates to assess branch support. Tips are coloured according to the breed. Abbreviations: LBG – Latvian Brown; LZG – Latvian Blue.
