# Supplementary material for: Genomic Characterization and Initial Insight into Mastitis-Associated SNP Profiles of Local Latvian Bos taurus Breeds
Source: Animals (Basel). 2023 Aug 31;13(17):2776. doi: 10.3390/ani13172776 (PMC10487150; doi:10.3390/ani13172776)
Supplement: Supplementary file 1 [file animals-13-02776-s001.zip › Figure S2.pdf]

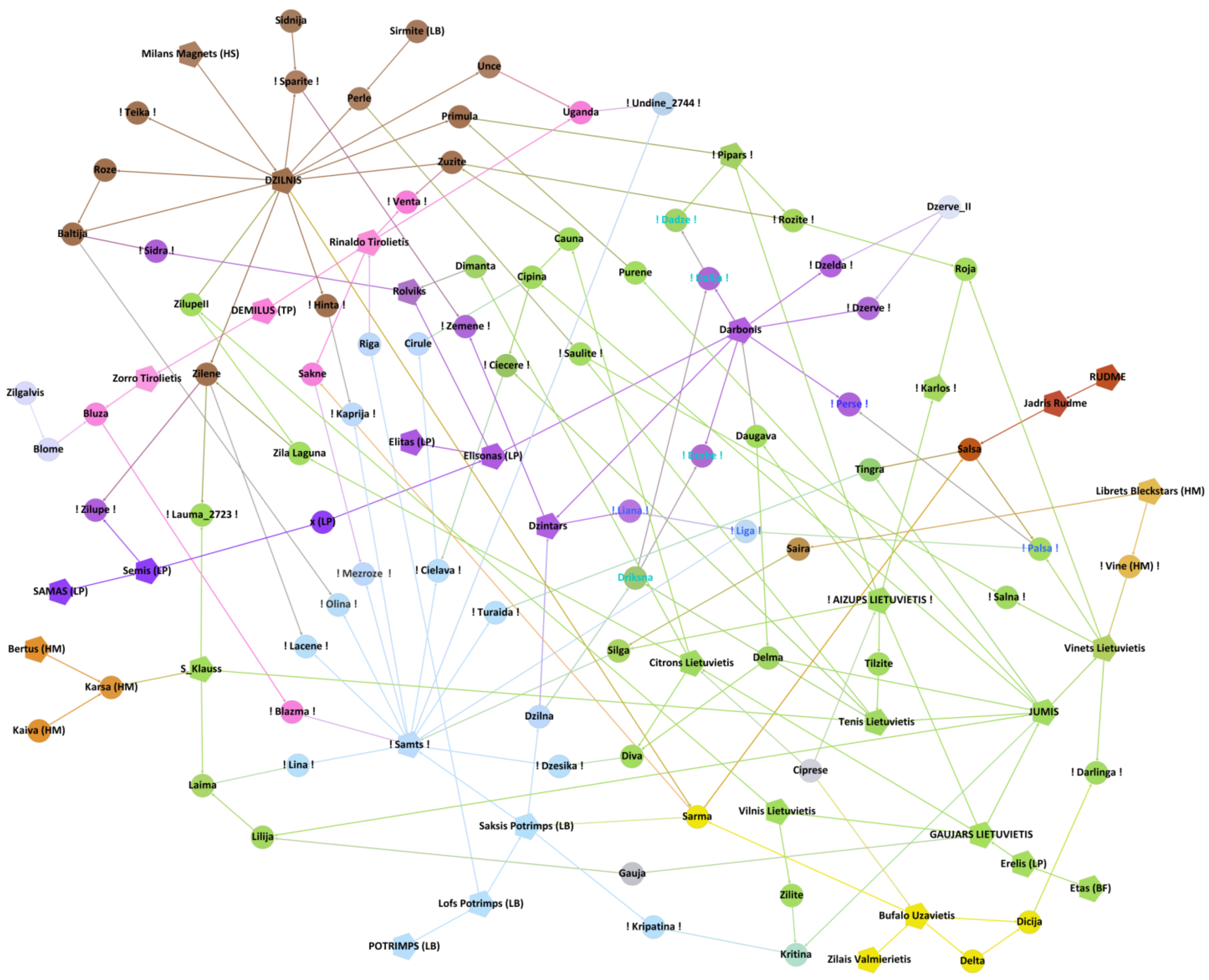

Supplementary Figure S2. Network showing kinship of LZG animals included in sequencing analysis. Dominant LZ paternal lineages are indicated by distinct colours of nodes and include lineage "Gaujars Lietuvietis" with two major branches "Aizups Lietuvietis" and "Jumis" both coloured in light green, "Dzilnis" in brown, "Bufalo Uzavietis" in yellow and "Librets Bleckstars" in orange. There is admixture of LB cattle through lineages "Rudme" and "Potrimps" coloured in red and blue respectively. Animals with ancestry of Tyrol Grey are coloured in pink, and Lithuanian Light Grey ancestry is indicated in violet. The male founder of each of the dominant lineages is denoted with capital letters. Maternal lineages with more than three sequenced cows has names of animals indicated in distinct colours and include sisters Durbe and Dalija along with Dadze daughter of Dalija (names in bright blue), and Palsa with daughter Liga and granddaughter Liana (names in dark blue).

Abbreviations in parenthesis indicate sires from other breeds including Latvian Brown cow (LB), Tyrol Grey (TP), Lithuanian Light Grey (LP), Holstein Black and White (HM) and Holstein Red (HS). "X" indicates animals with missing pedigree information. Exclamation marks indicate animals elected for sequencing analysis. Pentagons represent bulls and circles represent cows. Edges show relationships between animals and are directed from ancestors towards offspring. The length of edges is determined manually to provide better resolution and has no other meaning.
