# Supplementary material for: Genomic Characterization and Initial Insight into Mastitis-Associated SNP Profiles of Local Latvian Bos taurus Breeds
Source: Animals (Basel). 2023 Aug 31;13(17):2776. doi: 10.3390/ani13172776 (PMC10487150; doi:10.3390/ani13172776)
Supplement: Supplementary file 1 [file animals-13-02776-s001.zip › Figure S6.pdf]

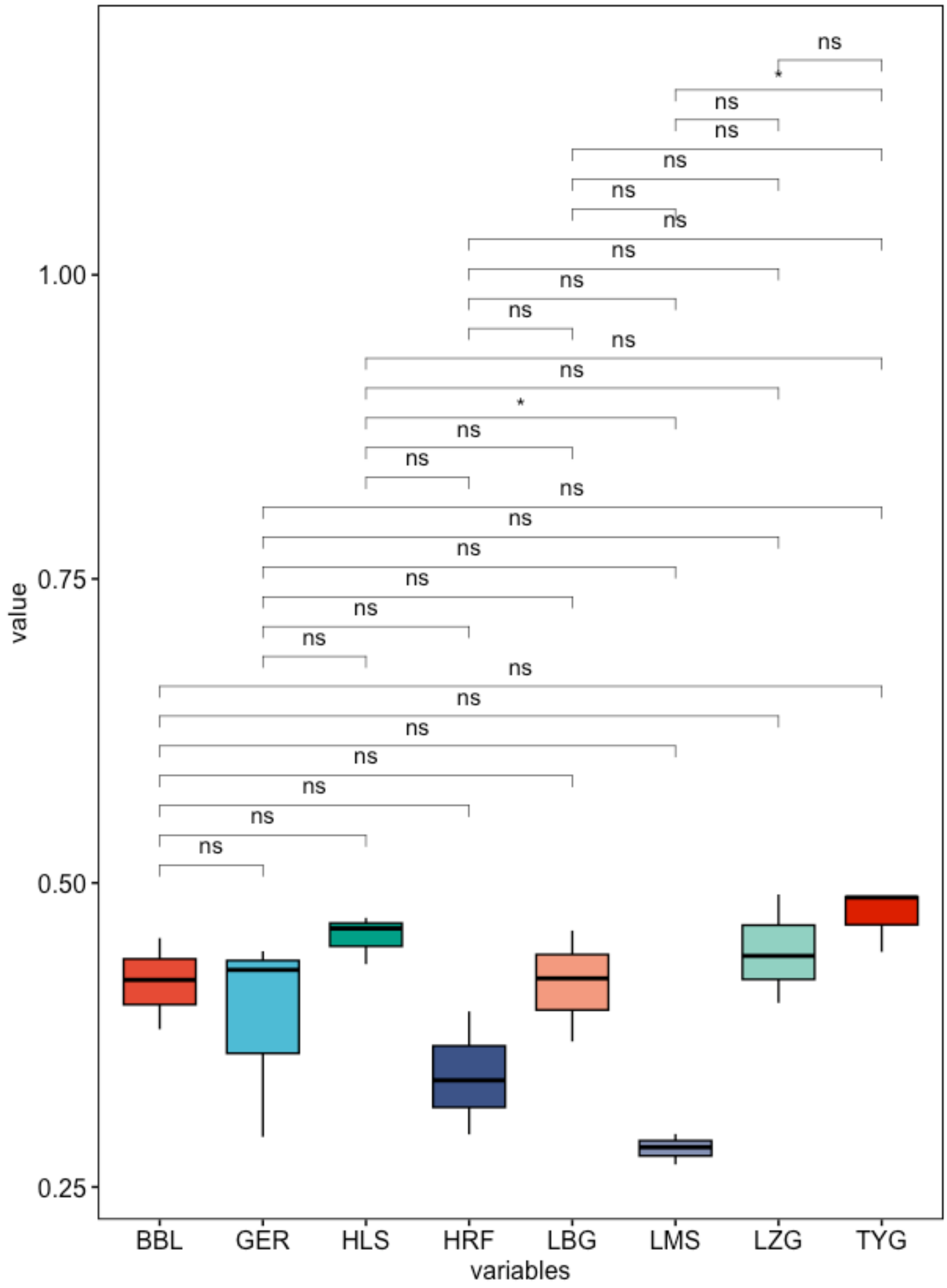

Supplementary Figure S 6. Genome heterozygosity for all breeds. To determine the significant differences between breed pairwise T-test with Bonferroni p-value adjustment was performed. Significance levels: 'ns' – non-significant, '*' – p = (0.01,0.05]. Abbreviations: BBL – Belgian Blue; GER – German angus; HLS – Holstein; HRF – Hereford; LBG – Latvian Brown; LMS – Limousin; LZG – Latvian Blue; TYG – Tyrolean grey.
