# Supplementary material for: Genomic Characterization and Initial Insight into Mastitis-Associated SNP Profiles of Local Latvian Bos taurus Breeds
Source: Animals (Basel). 2023 Aug 31;13(17):2776. doi: 10.3390/ani13172776 (PMC10487150; doi:10.3390/ani13172776)
Supplement: Supplementary file 1 [file animals-13-02776-s001.zip › Figure S9.pdf]

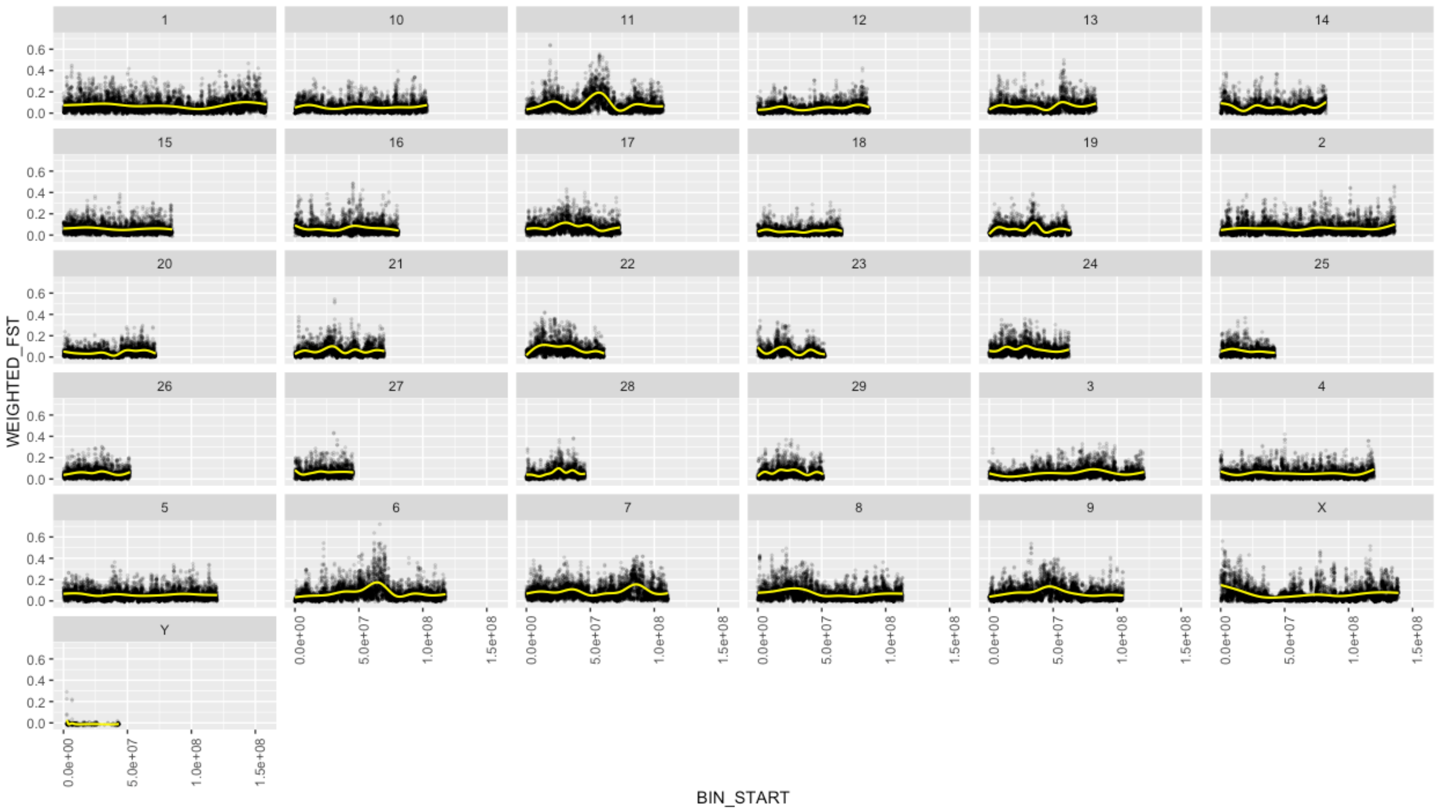

Supplementary Figure S9. Genome-wide distribution of weighted Fst between individuals of LZG and LBG breeds. High Fst value indicates a considerable degree of differentiation among the breeds.
