# Supplementary figures and images for: Genomic Characterization and Initial Insight into Mastitis-Associated SNP Profiles of Local Latvian Bos taurus Breeds
Source: Animals (Basel). 2023 Aug 31;13(17):2776. doi: 10.3390/ani13172776 (PMC10487150; doi:10.3390/ani13172776)

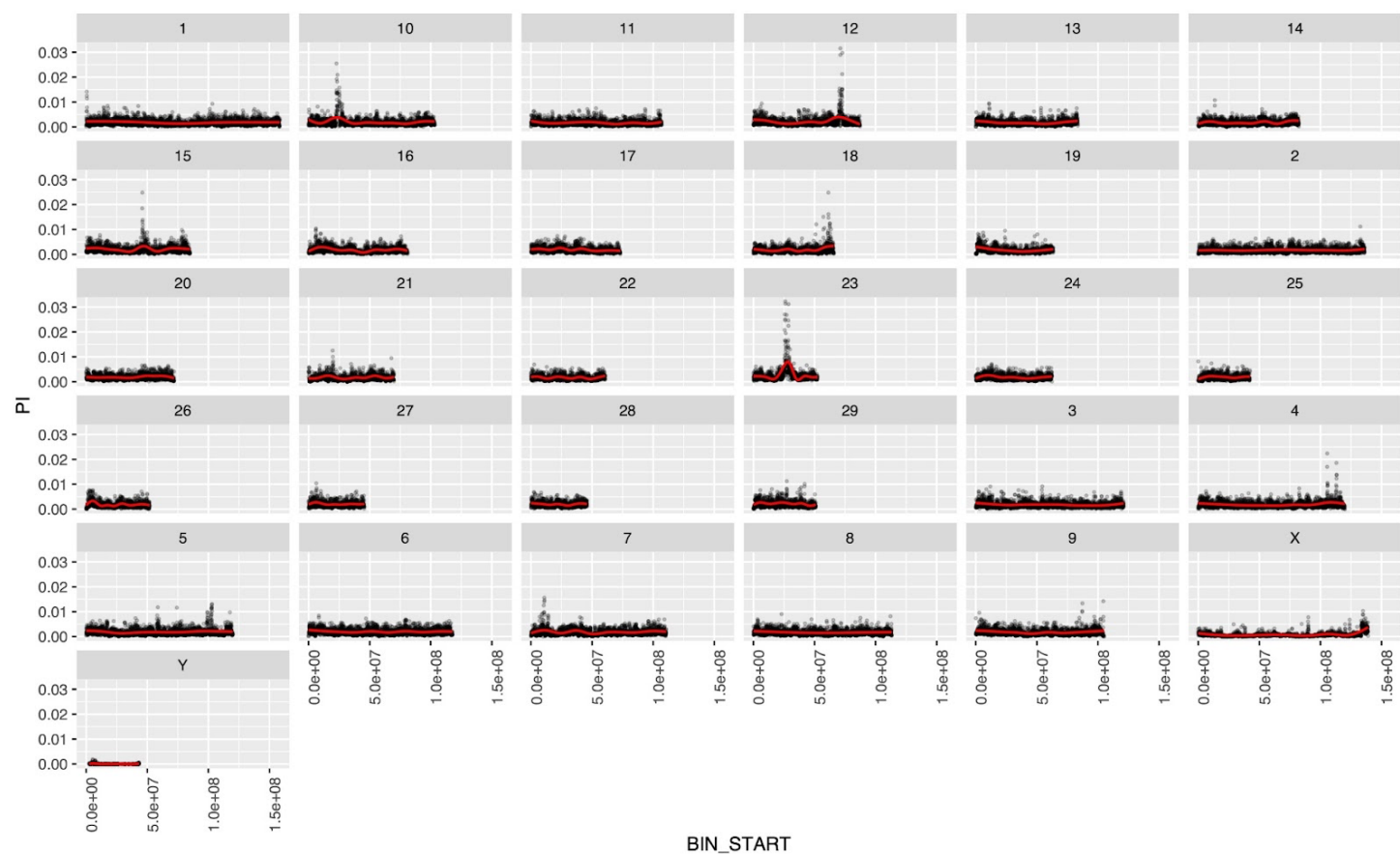

**Supplementary Figure S3.** Nucleotide divergence ( $\pi$ ) across all chromosomes of LBG breed (n=40).

Supplement: Supplementary file 1 [file animals-13-02776-s001.zip › Figure S3.pdf]

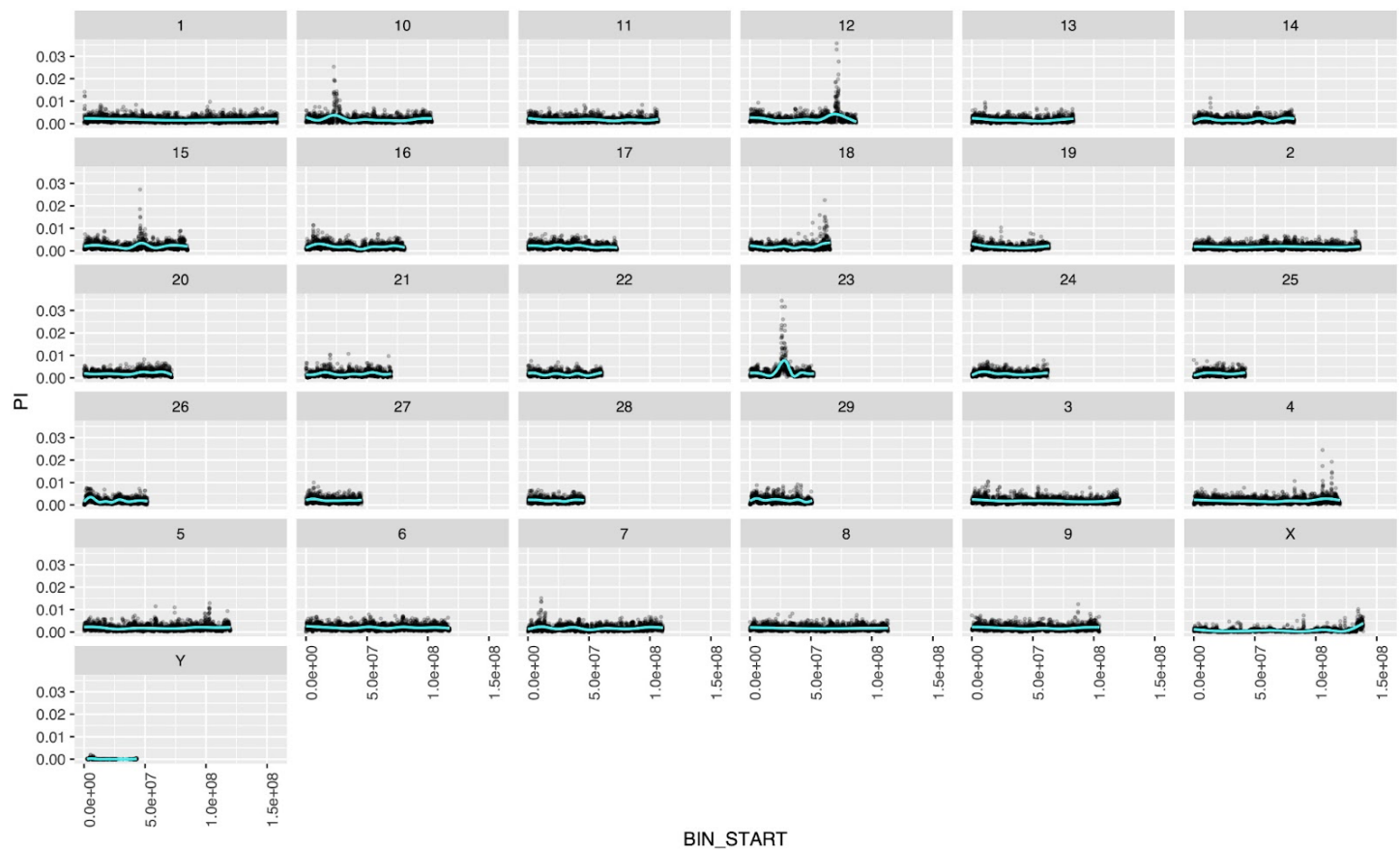

**Supplementary Figure S4.** Nucleotide divergence ( $\pi$ ) across all chromosomes of LZG breed (n=40).

Supplement: Supplementary file 1 [file animals-13-02776-s001.zip › Figure S4.pdf]

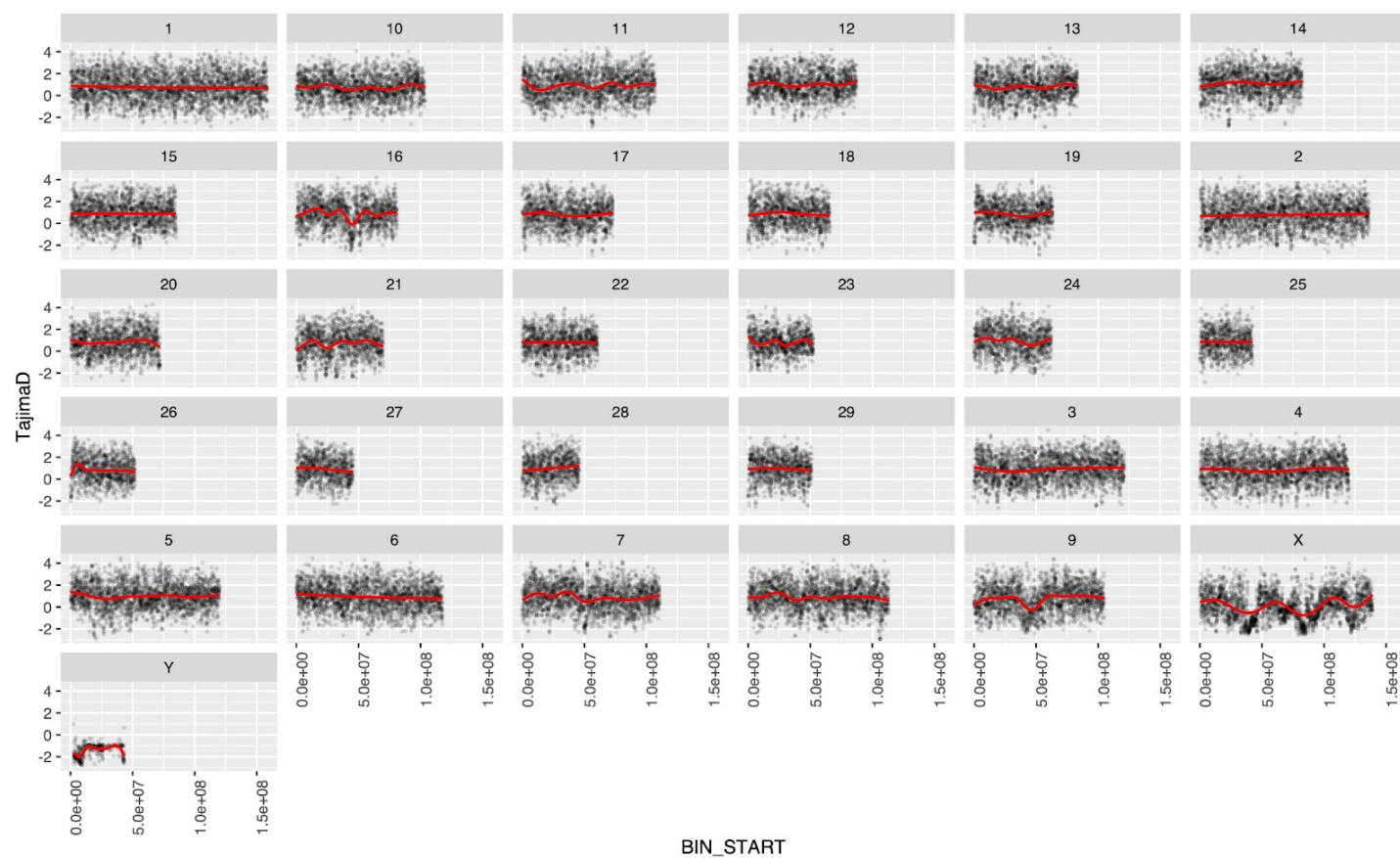

**Supplementary Figure S7.** Tajima's D across all chromosomes of LBG breed (n=40).

Supplement: Supplementary file 1 [file animals-13-02776-s001.zip › Figure S7.pdf]

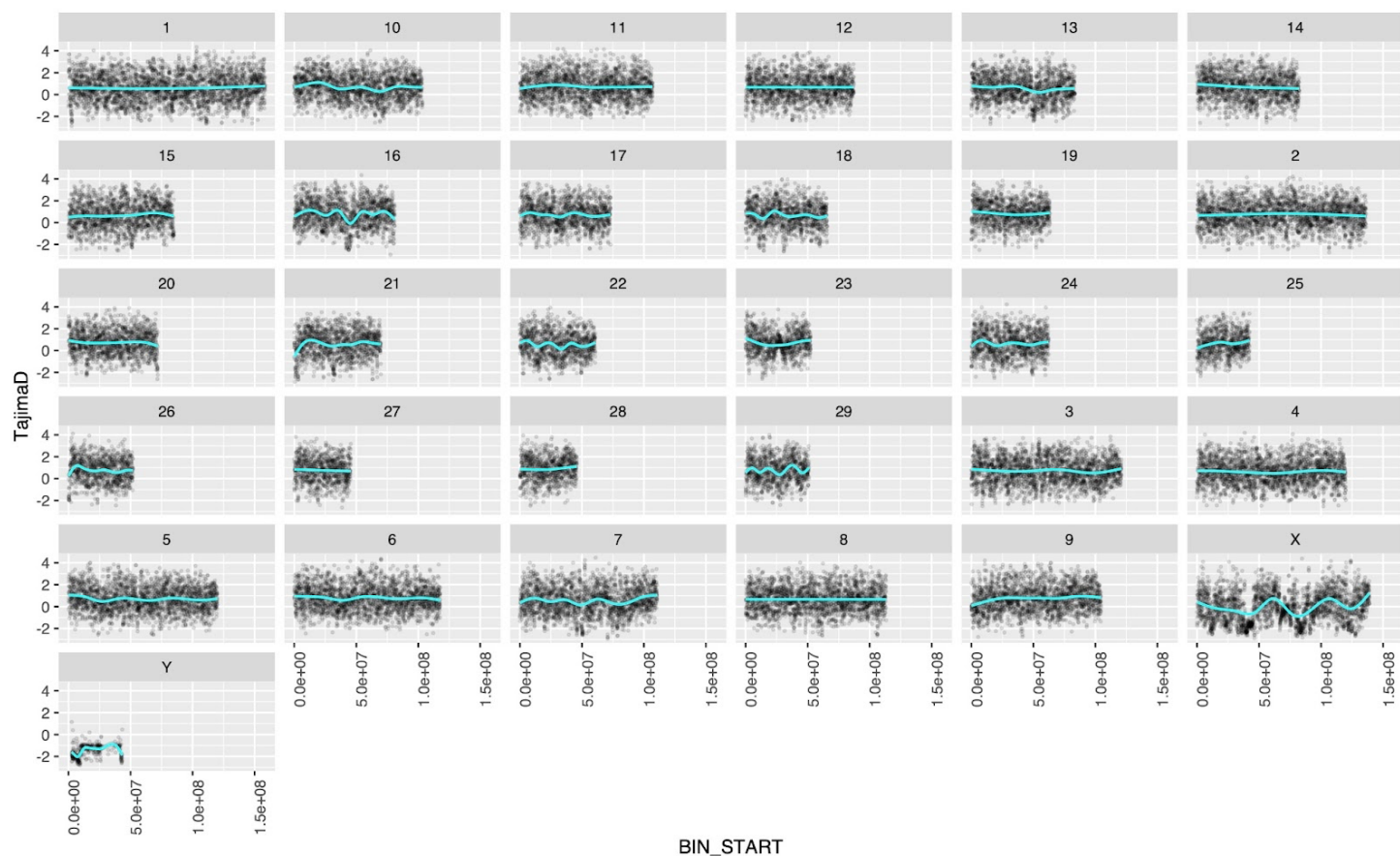

**Supplementary Figure S8.** Tajima's D across all chromosomes of LZG breed (n=40).

Supplement: Supplementary file 1 [file animals-13-02776-s001.zip › Figure S8.pdf]
